# Long-Term Outcomes and EUSOMA Quality Indicators in a Large Single-Center Surgical Breast Cancer Cohort from North Africa †

**DOI:** 10.3390/cancers18050731

**Published:** 2026-02-25

**Authors:** Amina Houmada, Halima Abahssain, Abdelilah Souadka, Amine Souadka

**Affiliations:** 1 Surgical Oncology Department, National Institute of Oncology, University Mohammed V in Rabat, Rabat 10000, Morocco; 2 Equipe de Recherche en Oncologie Translationnelle (EROT), Faculty of Medicine and Pharmacy of Rabat, University Mohammed V in Rabat, Rabat 10000, Morocco; 3 Oncology and Medical Specialties Department, Valenciennes General Hospital, 59300 Valenciennes, France; abahssainhalima@gmail.com; 4 Surgical Oncology Department, Private Oncology Center, Rabat 10000, Morocco; abdelilah.souadka@gmail.com

**Keywords:** breast cancer, long-term survival, North Africa, EUSOMA quality indicators, prognostic factors, targeted therapy access

## Abstract

**Background:** Long-term real-world data on breast cancer outcomes in North Africa remain limited, despite rising incidence and increasing access to multimodal treatment. This study reports survival outcomes, recurrence patterns, and quality-of-care performance in the largest single-center breast cancer cohort in the region. **Methods:** A retrospective analysis was conducted on a prospectively maintained registry of 1826 women who underwent curative-intent breast cancer surgery between 2002 and 2016. Overall survival (OS) and disease-free survival (DFS) were estimated using Kaplan–Meier methods. Prognostic factors were examined through univariate and multivariate Cox regression analysis. Adherence to selected EUSOMA quality indicators was assessed across two time periods. **Results:** At a median follow-up of 10 years, five-year OS and DFS were 96% and 90%, respectively, declining to 91% and 84% at 10 years. Local recurrence occurred in 6.2% of patients and distant metastasis in 11%, with bone being the most frequent metastatic site. TNM stage, tumor size, SBR grade, and tumor localization were independent predictors of OS, while younger age and TNM stage independently predicted DFS. Hormone receptor status and TNM stage were associated with local recurrence; age and tumor site predicted distant metastasis. Quality-of-care evaluation showed high adherence to EUSOMA indicators, including timeliness of surgery, proportion of single-operation resections, and use of postoperative radiotherapy after breast-conserving surgery. Limited administration of HER2-targeted therapy during the earlier years of the cohort reflected historical availability constraints rather than current practice. **Conclusions:** This study provides robust long-term evidence from North Africa, demonstrating that high survival rates can be achieved when standardized surgical pathways, multidisciplinary coordination, and adherence to quality indicators are maintained. The findings underline the importance of sustained investment in diagnostic access and treatment organization and highlight the expected benefits of the expanded availability of HER2-targeted therapies in the region. These results offer a valuable benchmark for strengthening breast cancer care in comparable LMIC settings.

## Simple Summary

Breast cancer is the most frequent cancer among women in North Africa, yet long-term survival data from the region remain scarce. This study followed 1826 Moroccan women who underwent surgery for breast cancer and provides one of the largest and longest evaluations of outcomes in the area. After 10 years of follow-up, survival rates were high and comparable to those reported in many high-income countries. Most patients were treated according to international standards, with excellent adherence to recommended timelines, surgical quality, radiotherapy, and endocrine therapy. The limited use of HER2-targeted therapies in earlier years reflects historical availability rather than current practice. Overall, these findings show that well-organized breast cancer care can achieve strong long-term outcomes even in resource-limited settings. They also highlight the importance of continuing to improve access to modern treatments to ensure that all patients benefit equally from advances in breast cancer therapy.

## 1. Introduction

Breast cancer is the most frequently diagnosed malignancy and the leading cause of cancer-related mortality among women worldwide. In 2020, 2.3 million women were diagnosed, and 685,000 deaths were attributed to the disease [1]. While survival has improved substantially over the past three decades owing to earlier detection and multimodal treatment strategies, these gains have not been evenly distributed across regions. In low- and middle-income countries (LMICs), limited access to screening, delays in diagnosis, and unequal availability of systemic therapies continue to influence outcomes and contribute to persistent global disparities [2].

In North Africa, the reported incidence is 29.3 per 100,000 women, a figure that is steadily increasing [3] driven by demographic changes and expanding access to imaging. Although diagnostic tools such as mammography, ultrasound, and, more recently, breast MRI have improved early detection [4]. However, despite these advances, access to timely diagnosis and treatment remains a challenge, with a large proportion of patients still presenting with locally advanced or metastatic disease at diagnosis [5]. Consequently, survival outcomes vary widely: five-year survival rates exceed 90% in high-income countries, compared to 66% in India and as low as 40% in sub-Saharan Africa [6], which reflects heterogeneity in healthcare infrastructure, treatment availability, and multidisciplinary coordination.

Concurrently, breast cancer management has evolved toward more personalized and less invasive approaches. Breast-conserving surgery, sentinel lymph node biopsy, targeted therapies, and standardized radiotherapy protocols have become integral components of modern care [7,8]. Molecular classification has further refined therapeutic strategies, identifying at least four subtypes with distinct prognoses and treatment approaches: Luminal A, Luminal B, HER2-positive, and triple-negative breast cancer (TNBC) [9].

Despite these advances, real-world data on long-term oncologic outcomes in North African populations remain scarce. The majority of survival data are derived from Western cohorts, with limited representation of African or Middle Eastern patients [10].

The implementation of international quality indicators—such as those defined by the European Society of Breast Cancer Specialists (EUSOMA)—has helped harmonize treatment pathways and provides a useful framework to evaluate the performance of breast cancer units. However, very few studies from LMIC settings have assessed adherence to these indicators or examined how quality-of-care metrics relate to long-term survival.

Against this background, the present study offers a comprehensive evaluation of breast cancer outcomes from one of the largest single-center cohorts in North Africa, comprising 1826 women treated surgically over a 15-year period. The objectives were to assess overall and disease-free survival, describe recurrence patterns, identify prognostic factors, and evaluate adherence to selected EUSOMA quality indicators across two time periods. By providing robust long-term data from a region where such evidence is limited, this study aims to contribute to a better understanding of breast cancer trajectories in LMIC settings and to highlight areas where improvements in access, quality, and equity of care could further enhance outcomes.

In this study, North Africa encompasses the Maghreb and neighboring countries, including Morocco, Algeria, Tunisia, and Egypt. Most published breast cancer series from this region are registry-based or multicenter studies with limited surgical detail and long-term follow-up.

## 2. Materials and Methods

### 2.1. Study Design and Setting

This study is a retrospective analysis of a prospectively maintained breast cancer registry. The cohort includes all women who underwent curative-intent surgery at a specialized oncology center in Morocco between January 2002 and December 2016. Multidisciplinary management followed European recommendations throughout the study period.

### 2.2. Patient Selection

Eligible patients had a histologically confirmed invasive breast carcinoma and underwent surgery with curative intent. Exclusion criteria were: (1) benign breast disease, (2) male breast cancer, and (3) absence of postoperative follow-up data. Only patients with updated survival status were included in the final survival analyses.

The main analytic cohort consisted exclusively of patients with invasive breast carcinoma. Cases of ductal carcinoma in situ (DCIS) were not included in survival or prognostic analyses. However, DCIS cases were retained in the database and used solely for descriptive analyses and for the calculation of EUSOMA quality indicators when explicitly required by the indicator definition. Indicator-specific denominators were applied accordingly, these indicators were defined according to the European Society of Breast Cancer Specialists recommendations (Del Turco et al., 2010; Biganzoli et al., 2017) [5,6].

### 2.3. Follow-Up Procedures

Follow-up information was obtained through scheduled outpatient clinic visits and, when necessary, structured telephone contact with patients or their relatives. Survival status, date of death when applicable, and recurrence events were recorded prospectively in the institutional database.

Patients without documented recurrence or death were censored at the date of their last confirmed contact. Loss to follow-up increased with longer follow-up duration, reflecting the long study period and patient mobility.

### 2.4. Data Collection and Variables

Demographic, clinicopathologic, treatment, and outcome data were collected prospectively. Variables included tumor characteristics (histologic type, pT, pN, SBR grade, hormone receptor status, HER2 status, Ki-67 when available), type of surgery, neoadjuvant and adjuvant treatments, and recurrence patterns. Proportional hazards assumptions were assessed using standard methods and were not materially violated. Missing data were handled using complete-case analysis. The proportion of missing values for key variables is reported, and the number of patients included in each multivariable model is specified.

Outcomes were defined as follows:

- Primary outcome: Overall survival (OS);
- Secondary outcomes: Disease-free survival (DFS), local recurrence, distant metastasis;
- Quality-of-care outcomes: adherence to selected European Society of Breast Cancer Specialists (EUSOMA) indicators across two time periods: 2002–2008 and 2009–2016, reflecting the progressive implementation of modern diagnostic and surgical techniques.

### 2.5. Outcome Definitions

OS was defined as the time from surgery to death from any cause. DFS was defined as the interval from surgery to local, regional, or distant recurrence. Local recurrence included ipsilateral breast, chest wall, or regional lymph nodes; distant metastasis included bone, liver, lung, brain, or other distant sites. At longer follow-up horizons, an increasing proportion of patients were censored due to loss to follow-up, particularly beyond 10 years. These patients were treated as censored observations in survival analyses.

### 2.6. Statistical Analysis

Survival probabilities were estimated using the Kaplan–Meier method and compared with the log-rank test. Variables associated with survival in univariate analysis (*p* < 0.20) were entered into a multivariate Cox proportional hazards model with backward stepwise selection applied to derive parsimonious models. Cox proportional hazards regression was used to estimate hazard ratios (HR) and 95% confidence intervals (CI). Recurrence and distant metastasis were analyzed using Kaplan–Meier methods. Death was treated as a censoring event; therefore, competing risks were not accounted for, and cumulative event probabilities may be overestimated. Missing data were handled by complete-case analysis. Statistical significance was set at *p* < 0.05.

Variables associated with outcomes in univariable analysis (*p* < 0.20) were considered for multivariable Cox proportional hazards models. To derive parsimonious and clinically interpretable models, a backward stepwise selection procedure was applied. To avoid collinearity, staging variables were handled a priori: TNM stage was not entered simultaneously with its components (pT and pN). Multivariable models were therefore constructed using either TNM stage or pT/pN, but not both. Results are reported as hazard ratios (HR) with 95% confidence intervals (CI).

EUSOMA quality indicators were calculated using indicator-specific denominators, in accordance with published EUSOMA definitions. Conditional indicators were applied as required, including restriction to invasive carcinoma or DCIS-only populations, exclusion of patients receiving primary systemic therapy, and tumor size or nodal status thresholds.

Analyses were performed using SPSS v25.

### 2.7. Ethical Considerations

The study was approved by the institutional review board (CIC—University Hospital Ibn Sina) in accordance with Moroccan law (Law 28/13–Article 2). Additional approvals were obtained from the local ethics committees of the National Institute of Oncology and collaborating centers. All patients provided informed consent at the time of data entry into the registry.

## 3. Results

### 3.1. Baseline Characteristics

A total of 1826 women were included. The mean age was 51 years, and 86% were older than 40. Most tumors were unilateral (97.2%) and unifocal (88.1%). Breast-conserving surgery was performed in 57.2% of patients, while 42.8% underwent mastectomy. Invasive carcinoma of no special type was the predominant histologic subtype (79.2%). Nearly half of all tumors were classified as pT2 (49.6%), and 39.6% were node-negative. Hormone receptors were positive in 72.6% of women, HER2 was amplified in 16%, the median Ki-67 index was 23.5%, and 47.1% of tumors were classified as Luminal A.

Adjuvant chemotherapy was administered to 76.7% of patients, radiotherapy to 86%, endocrine therapy to 72.6%, and Trastuzumab to 16% of HER2-positive patients, reflecting the limited availability of targeted therapy during the earlier years of the cohort (Table 1).

### 3.2. Survival Outcomes

**Figure 1:**
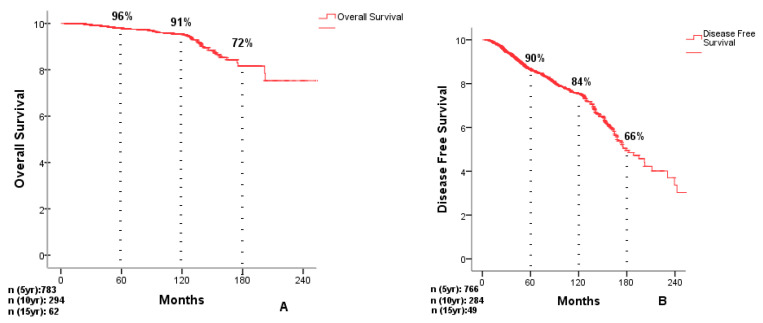
Overall survival curve (**A**). Disease-free survival curve (**B**).

At a median follow-up of 10 years, the five-year overall survival (OS) was 96%, decreasing to 91% at 10 years and 72% at 15 years. Disease-free survival (DFS) was 90% at five years, 84% at 10 years, and 66% at 15 years. Survival curves are presented in Figure 1.

**Figure 2:**
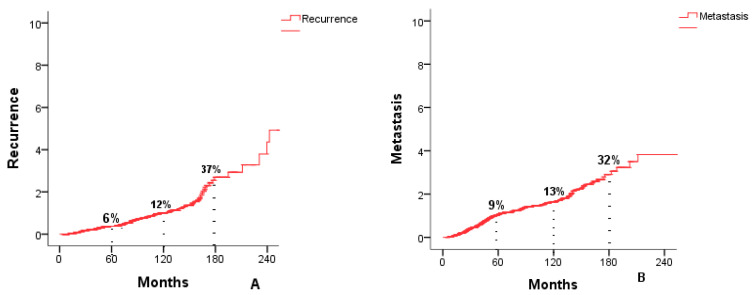
Recurrence occurrence curve (**A**). Metastasis occurrence Curve (**B**).

The occurrence of local and locoregional recurrence was found in 114 patients, at 1 year it was at 0.4%, at 5 years and at 10 years it was at 6% and 12%, respectively. The occurrence of metastasis was found in 202 patients, at one year it was 1.7%, at 5 years and at 10 years it was at 9% and 13%, respectively. Survival estimates beyond 10 years are based on a limited number of patients and should therefore be interpreted with reduced precision, particularly for cases diagnosed in the later years of the cohort. Survival curves are presented in Figure 2.

### 3.3. Local Recurrence and Distant Metastases

Local or locoregional recurrence occurred in 114 patients (6.2%). Cumulative recurrence was 0.4% at one year, 6% at five years, and 12% at 10 years. Among women treated with breast-conserving surgery, 60% of recurrences occurred in the ipsilateral breast; lymph-node or cutaneous recurrences represented 57% of cases.

Distant metastasis was documented in 202 patients (11%). Incidence reached 1.7% at one year, 9% at five years, and 13% at 10 years. The bone was the most common metastatic site (33.7%), followed by lung (15.2%), liver (14.6%), and brain (7%). Multiple organ involvement was observed in 29.5% of metastatic cases. Treatment decisions were discussed in multidisciplinary tumor boards, with systemic therapy recommended in 57% of patients, which may not always correspond to treatment actually received due to clinical condition, patient preference, or access-related constraints.

### 3.4. Predictive Factors for Survival

In univariate analysis, overall survival was associated with tumor localization, vascular emboli, pT stage, SBR grade, and TNM stage. Multivariate Cox analysis identified four independent predictors of OS: tumor localization (*p* = 0.003), pT classification (*p* = 0.017), SBR grade (*p* = 0.025), and TN M stage (*p* = 0.004) (Table 2).

For disease-free survival, younger age (*p* = 0.021) and TNM stage (*p* < 0.001) remained significant independent predictors (Table 3).

### 3.5. Predictors of Local Recurrence and Metastasis

Local recurrence was independently associated with hormone receptor status (*p* = 0.007) and TNM stage (*p* = 0.009). Metastasis was significantly associated with age at diagnosis (*p* = 0.046), tumor site (*p* = 0.014), and TNM stage (*p* < 0.001) (Table 4 and Table 5).

### 3.6. Quality-of-Care Indicators

Evaluation of EUSOMA quality indicators showed high adherence. selected process- and treatment-related indicators. Preoperative histologic confirmation increased from 22.2% to 37.1% across the two study periods, while timely surgery within six weeks was achieved in more than 97% of patients. Nearly all invasive tumors (98%) were treated with a single breast operation. Postoperative radiotherapy after breast-conserving surgery was administered in 92% of cases, exceeding EUSOMA minimum standards. However, several diagnostic and staging-related indicators did not meet EUSOMA minimum standards, particularly during the early years of the study period such as the use of Trastuzumab that met the contextual constraints of the study period and has since improved substantially.

## 4. Discussion

Breast cancer management has undergone profound changes over the past two decades, and the trajectories of survival reported worldwide increasingly reflect the integration of multimodal therapy, improved diagnostic pathways, and stronger quality-of-care frameworks. In this context, the present study provides one of the most comprehensive long-term analyses from North Africa, covering 1826 women treated surgically over a 15-year period. The principal finding is that overall survival and disease-free survival at 5 and 10 years mirror outcomes reported in several high-income settings, despite important differences in healthcare resources and limited access to targeted systemic therapies. Such results are noteworthy and challenge the assumption that long-term oncologic outcomes in low- and middle-income countries (LMICs) are universally inferior (Tables S1–S3).

### 4.1. Comparison with Existing Literature

Breast cancer is age-dependent, in our study the median age of patients was 51 years, similar to the peak age of diagnosis for African and Asian women [9] compared to 60–70 years in western countries [10]. Many studies have shown that unilateral breast cancer is more frequent in the left breast than in the right [11] confirmed by subsequent studies showing even the involvement of tumor site as a prognostic factor [12]. Neoadjuvant chemotherapy is currently a powerful tool in downstaging and converting locally advanced and inoperable tumors to operable ones [13], in our study the patients had neoadjuvant chemotherapy mainly for locally advanced tumors.

In our survey luminal A tumors were predominant representing 47.1%, similar to some results reported in Moroccan studies [14,15] and also in American and European countries [16,17], a higher porpotion of triple negative tumors and a lower one for HER2+ subtypes has been observed compared to other Moroccan and European countries [15,18,19]. All the patients with positive Hormone receptors and/or HER2 receptor amplification had hormonotherapy and targeted therapy.

### 4.2. Survival Outcomes in Context

The five-year overall survival of 96% and the ten-year survival of 91% observed in this cohort rank among the highest published in real-world series. compared to survival rates that vary tremendously by region, from an overall 5- year survival at 90% and 88.1% in the USA and Germany respectively [20], with a 10-year relative survival rate at 83% in a report of the American Cancer Society [21] to 3-, 5- and 10-year survival rate of women with breast cancer in the Eastern Mediterranean Region with 80% and 71% and 56% respectively [22] and a 5-year survival rate varied from 52% in India to 82% in China [23]. Several factors likely contribute to these outcomes. First, the median age of 51 years aligns with the demographic profile reported across Africa and Asia, where breast cancer tends to occur at younger ages. Younger patients often have fewer comorbidities, which may contribute to improved postoperative recovery and treatment tolerance. Second, early surgical management and adherence to recommended timelines—both critical determinants of long-term outcome—were consistently maintained across the study period. Finally, the predominance of hormone-receptor-positive disease (nearly 75%) may partly explain the favorable long-term prognosis, particularly given the high rates of adherence to endocrine therapy.

It should be noted that survival estimates beyond 10 years rely on a relatively small number of patients and are therefore subject to reduced precision, especially for cases diagnosed in the later years of the cohort.

### 4.3. Patterns of Recurrence and Prognostic Determinants

Local recurrence (6.2%) and distant metastasis (11%) occurred at rates consistent with large international cohorts. Bone metastases, the most frequent site of distant relapse, accounted for one-third of cases, reflecting the typical metastatic pattern of hormone-receptor-positive tumors. Multivariate analyses confirmed the central prognostic role of TNM stage across all survival dimensions. Tumor size, histologic grade, and lymph node involvement remained strong determinants of overall survival, underscoring the continued relevance of classical clinicopathologic staging even in an era of molecular stratification.

Interestingly, age and receptor status influenced recurrence patterns differently. Younger age independently predicted shorter disease-free survival, an observation frequently attributed to a higher prevalence of biologically aggressive tumors within this subgroup. Conversely, hormone receptor positivity was protective against local recurrence, while HER2 status did not retain statistical significance in multivariable models—likely reflecting the limited availability of trastuzumab during the earlier years of the cohort. These findings highlight the need for equitable access to targeted agents in the region, as insufficient anti-HER2 coverage may attenuate survival gains otherwise achieved.

### 4.4. Alignment with Global Literature

Several observations from this cohort resonate with international trends. The predominance of Luminal A tumors is consistent with reports from both African and Western populations and supports the notion that hormone-receptor-positive disease remains the backbone of breast cancer epidemiology globally. The distribution of molecular subtypes, the relatively low share of triple-negative tumors, and the decreasing rates of advanced locoregional disease over time all align with global patterns observed following expansion of screening and earlier diagnostic access.

What distinguishes the present cohort is not the distribution of tumor biology, but rather the ability to achieve high long-term survival in a resource-limited environment. This raises an important point for breast cancer care in LMICs: organizational quality and adherence to evidence-based care pathways may offset—in part—the lack of universal access to costly systemic innovations. The magnitude of this effect, however, should not obscure the need to address inequities in access to anti-HER2 treatments and next-generation targeted therapies, which remain critical components of modern breast cancer management.

### 4.5. Strengths and Implications

Beyond its size and follow-up duration, the study’s main strength lies in its integration of survival analysis with quality-of-care assessment. Few LMIC studies have simultaneously examined outcomes and the processes that shape them. The findings illustrate that improvements in diagnostic pathways, surgical standardization, and multidisciplinary management can produce survival outcomes comparable to high-income settings, even in the absence of comprehensive access to novel therapeutics. This has implications for national cancer-control strategies, particularly in regions where investments in operating-room capacity, radiotherapy, and early diagnosis may yield substantial gains while systemic therapy access continues to evolve.

The survival outcomes observed in this cohort are encouraging and fall within the range reported by several contemporary series from high-income countries. However, these comparisons should be interpreted with caution, given important differences in study design, patient selection, and healthcare organization.

## 5. Study Limitations

Several limitations should be considered when interpreting these findings. Although the database was prospectively maintained, the retrospective design remains subject to selection and information bias. This is particularly relevant for the earlier years of the cohort, when diagnostic and documentation practices were still evolving. The single-center nature of the study may also limit generalizability, although the large sample size and standardized treatment protocols mitigate this concern.

Incomplete availability of some clinicopathologic variables, particularly HER2 status, Ki-67, and TNM stage in earlier years, may have introduced bias through complete-case analysis and reduced the effective sample size of multivariable models. This limited the depth of subtype-specific analyses. Loss to follow-up increased with longer survival horizons, potentially leading to underestimation of late recurrences or metastases despite repeated attempts to update patient status.

Restricted access to targeted agents—most notably trastuzumab—represents an important contextual limitation and may have tempered long-term outcomes for HER2-positive patients. This therapeutic inequity is a persistent challenge in many LMIC settings and underscores the need for broader access to modern systemic therapies.

The quality-of-care analysis highlights a dual reality: while several core treatment indicators reached or exceeded EUSOMA minimum standards, important gaps persisted in diagnostic confirmation, sentinel lymph node utilization, and DCIS management. These findings reflect the stepwise implementation of breast cancer care infrastructure in a resource-limited setting and underscore the need to interpret EUSOMA indicators within their temporal and contextual framework.

Follow-up relied on a combination of outpatient visits and telephone contact, which may have led to under-ascertainment of late recurrences or metastatic events, particularly among patients lost to follow-up. In addition, loss to follow-up increased with longer survival horizons, potentially leading to an overestimation of survival outcomes. These limitations should be considered when interpreting long-term results. We also need to mention that Because death was treated as non-informative censoring in recurrence and metastasis analyses, the Kaplan–Meier method may overestimate cumulative incidence. Competing-risk approaches such as Fine–Gray models could provide more accurate estimates and should be considered in future studies.

This study was conducted in a single specialized oncology center, which may introduce referral and selection bias. Patients treated in such centers may benefit from earlier diagnosis, more standardized surgical pathways, and closer multidisciplinary follow-up than the general breast cancer population.

## 6. Conclusions

This study provides one of the most comprehensive long-term assessments of breast cancer outcomes in North Africa and demonstrates that survival rates can be comparable to those achieved in high-income countries when care is delivered within structured, multidisciplinary pathways. The high adherence to EUSOMA indicators—particularly regarding diagnostic confirmation, surgical quality, and appropriate use of radiotherapy and endocrine therapy—highlights the impact of organizational standards on long-term disease control.

The limited use of HER2-targeted therapy observed in this cohort reflects the historical context of the study period rather than current practice. Access to trastuzumab and other anti-HER2 agents has expanded significantly in recent years across the region, and future cohorts are expected to show improved outcomes for HER2-positive disease as a result. This transition illustrates how the progressive availability of modern systemic therapies can further consolidate survival gains already achieved through high-quality surgical and radiotherapeutic care.

Beyond its regional contribution, this cohort offers a valuable benchmark for health systems in low- and middle-income settings. It shows that strengthening diagnostic pathways, ensuring timely treatment, and standardizing surgical and multidisciplinary care can markedly narrow the survival gap with high-resource environments—even before the widespread adoption of newer biologic treatments. Future research should assess how the integration of contemporary targeted and molecular therapies, together with enhanced access to genetic and biologic profiling, will shape the next decade of breast cancer outcomes in North Africa.

## Figures and Tables

**Table 1:** Baseline clinicopathologic features of patients.

Parameter	Frequency 1826 (%)
Age	
<40 years	254 (13.9%)
>40 years	1572 (86.1%)
Laterality	
Right Breast	829 (45.4%)
Left Breast	881 (48.3%)
Both Breasts	52 (2.8%)
Unknown	64 (3.5%)
Number Of Tumor	
Unifocal	1609 (88.1%)
Multifocal	217 (11.9%)
Quadrant Distribution	
QSE	569 (31.1%)
Other Quadrant	775 (42.5%)
Unknown	482 (26.4%)
Biopsy	
Yes	549 (30.1%)
No	1277 (69.9%)
Neoadjuvant Chemotherapy	
Yes	125 (6.8%)
No	1701 (93.2%)
Surgery	
Conservative	1044 (57.2%)
Radical	782 (42.8%)
Lymph Node Dissection	1686 (92.3%)
Yes	140 (7.7%)
No	
Histological Type	
Invasive carcinoma with No special Type (NST)	1447 (79.2%)
Invasive Lobular Carcinoma	103 (5.6%)
Other	276 (15.2%)
pT Classification	
pT1	479 (26.2%)
pT2	906 (49.6%)
pT3	179 (9.8%)
pT4	13 (0.7%)
pTx	250 (13.7%)
pN Classification	
pN0	724 (39.6%)
pN1	400 (21.9%)
pN2	293 (16%)
pN3	219 (12%)
Unknown	190 (10.4%)
Intraductal Component	
Yes	890 (48.7%)
No	936 (51.3%)
Vascular Emboli	
Yes	496 (27.2%)
No	1330 (72.8%)
Hormonal Receptor	
Positive	1326 (72.6%)
Negative	391 (21.4%)
Unknown	109 (6%)
HER-2	
Positive	292 (16%)
Negative	1109 (60.7%)
Unknown	425 (23.3%)
Ki67	
Yes	677 (37.1%)
No	1149 (62.9%)
Biomolecular Classification	
Luminal A	861 (47.1%)
Luminal B	207 (11.3%)
HER2+	84 (4.6%)
Triple Negative	241 (13.2%)
Unknown	434 (23.8%)
Chemotherapy	
Yes	1400 (76.7%)
No	357 (19.5%)
Unknown	69 (3.8%)
Radiotherapy	
Yes	1404 (86%)
No	194 (11.9%)
Unknown	34 (2.1%)
Hormonotherapy	
Yes	1326 (72.6%)
No	391 (21.4%)
Unknown	109 (6%)
Targeted Therapy (Herceptin)	
Yes	292 (16%)
No	1534 (84%)
TNM Stage	
0	67 (3.7%)
Ia	227 (12.4%)
IIa	425 (23.3%)
IIb	236 (12.8%)
IIIa	252 (13.9%)
IIIb	3 (0.2%)
Iv	29 (1.6%)
Unknown	587 (32.2%)
Relapse	
Yes	114 (6.2%)
No	1712 (93.8%)
Metastasis	
Yes	202 (11%)
No	1624 (89%)

**Table 2:** Univariate and multivariate analysis for overall survival.

Variables	Univariate Analysis			Multivariate Analysis		
	HR	IC 95%	*p*	HR	IC 95%	*p*
**age** <40 Yrs (Reference)	0.666	0.274–1.623	0.372	-	-	-
**laterality** Unilateral (Reference)	2.576	1.274–5.212	**0.008**	2.246	1.307–3.860	**0.003**
**tumor Number** Unifocal (Reference)	1.308	0.573–2.987	0.524	-	-	-
**neoadjuvant CT** Yes (Reference)	0.742	0.226–2.435	0.622	-	-	-
**surgery** Conservative (Surgery)	2.018	0.978–4.168	0.218	-	-	-
**vascular Emboli** Yes (Reference)	1.863	0.898–3.866	**0.095**	1.857	0.957–3.604	0.067
**pT class** pT1 (Reference)	2.058	1.143–3.707	**0.016**	1.902	1.123–3.220	**0.017**
**pN** pN0 (Reference)	1.255	0.440–3.580	0.672	-	-	-
**RH** Positive (Reference)	1.849	0.167–20.417	0.616	-	-	-
**HER2** Positive (Reference)	0.507	0.204–1.260	**0.144**	0.803	0.384–1.678	0.559
**SBR** SBR I (Reference)	1.933	1.038–3.599	**0.038**	1.905	1.085–3.344	**0.025**
**biomolecular classification** Luminal A (Reference)	1.367	0.552–3.386	0.5	-	-	-
**TNM stage** Stage I (Reference)	0.110	0.032–0.467	**0.006**	0.145	0.036–0.359	**0.004**

**Table 3:** Univariate and multivariate analysis for disease-free survival.

Variables	Univariate Analysis			Multivariate Analysis		
	HR	IC 95%	*p*	HR	IC 95%	*p*
**age** <40 Yrs (Reference)	0.595	0.374–0.947	**0.029**	0.647	0.447–0.936	**0.021**
**laterality** Unilateral (Reference)	1.103	0.779–1.561	0.580	-	-	-
**tumor Number** Unifocal (Reference)	0.938	0.572–1.535	0.798	-	-	-
**neoadjuvant CT** Yes (Reference)	0.861	0.430–1.727	0.674	-	-	-
**surgery** Conservative (Surgery)	1.184	0.815–1.719	0.376	-	-	-
**vascular emboli** Yes (Reference)	1.308	0.895–1.912	**0.165**	1.187	0.885–1.592	0.252
**pT class** pT1 (Reference)	1.264	0.23–1.732	**0.144**	1.200	0.956–1.507	0.116
**pN** pN0 (Reference)	1.332	0.772–2.297	0.302	-	-	-
**RH** Positive (Reference)	0.670	0.208–2.155	0.502	-	-	-
**HER2** Positive (Reference)	0.916	0.581–1.444	0.705	-	-	-
**SBR** SBR I (Reference)	1.004	0.744–1.354	0.982	-	-	**-**
**biomolecular classification** Luminal A (Reference)	0.911	0.579–1.435	0.689	-	-	-
**TNM stage** Stage I (Reference)	0.054	0.016–0.09	**<0.001**	0.107	0.013–0.095	**<0.00**

**Table 4:** Univariate and multivariate analysis for relapse occurrence.

Variables	Univariate Analysis			Multivariate Analysis		
	HR	IC 95%	*p*	HR	IC 95%	*p*
**age** <40 Yrs (Reference)	0.549	0.269–1.120	**0.099**	0.674	0.374–1.216	0.190
**laterality** Unilateral (Reference)	0.887	0.499–1.576	0.887	-	-	**-**
**tumor number** Unifocal (Reference)	0.677	0.255–1.793	0.432	-	-	**-**
**neoadjuvant CT** Yes (Reference)	1.927	0.619–5.995	**0.258**	0.691	0.302–1.577	0.379
**surgery** Conservative (Surgery)	0.747	0.392–1.424	0.375	-	-	-
**vascular emboli** Yes (Reference)	1.224	0.651–2.303	0.531	-	-	-
**pT class** pT1 (Reference)	1.248	0.718–2.169	0.433	-	-	-
**pN** pN0 (Reference)	1.341	0.558–3.218	0.512	-	-	-
**RH** Positive (Reference)	0.434	0.090–2.094	**0.299**	0.515	0.318–0.837	**0.007**
**HER2** Positive (Reference)	1.612	0.849–3.062	**0.145**	1.327	0.793–2.223	0.282
**SBR** SBR I (Reference)	0.998	0.607–1.641	0.994	-	-	**-**
**biomolecular classification** Luminal A (Reference)	0.884	0.466–1.678	0.707	-	-	-
**TNM stage** Stage I (Reference)	0.410	0.101–1.669	**0.213**	0.291	0.077–1.104	**0.009**

**Table 5:** Univariate and multivariate analysis for metastasis occurrence.

Variables	Univariate Analysis			Multivariate Analysis		
	HR	IC 95%	*p*	HR	IC 95%	*p*
**age** <40 Yrs (Reference)	0.553	0.319–0.957	**0.034**	0.660	0.439–0.993	**0.046**
**laterality** Unilateral (Reference)	2.046	0.600–6.982	0.253	2.548	1.213–5.534	0.014
**tumor number** Unifocal (Reference)	0.994	0.558–1.770	0.984	-	-	-
**neoadjuvant CT** Yes (Reference)	0.785	0.368–1.676	0.532	-	-	-
**surgery** Conservative (Surgery)	1.349	0.860–2.116	**0.193**	1.050	0.763–1.446	0.763
**vascular emboli** Yes (Reference)	1.064	0.672–1.683	0.792	-	-	-
**pT class** pT1 (Reference)	0.982	0.684–1.411	0.924	-	-	-
**pN** pN0 (Reference)	1.417	0.708–2.835	0.325	-	-	-
**RH** Positive (Reference)	1.333	0.307–5.785	0.701	-	-	-
**HER2** Positive (Reference)	0.931	0.523–1.657	0.807	-	-	-
**SBR** SBR I (Reference)	0.912	0.634–1.312	0.620	-	-	-
**biomolecular classification** Luminal A (Reference)	1.054	0.592–1.875	0.858	-	-	-
**TNM stage** Stage I (Reference)	0.058	0.018–0.183	**<0.001**	0.037	0.014–0.099	**<0.001**

## Data Availability

The original contributions presented in this study are included in the article. Further inquiries can be directed to the corresponding authors.

## Supplementary Materials

The following supporting information can be downloaded at: https://www.mdpi.com/article/10.3390/cancers18050731/s1, Table S1: Correlation of molecular subtypes with prognostic factors; Table S2: A Two-Period Comparison of Quality Indicators in Breast Cancer Management; Table S3: Comparative table describing baseline clinicopathologic characteristics of patients included vs excluded in the survival analysis.
