# Supplementary material for: Long-Term Outcomes and EUSOMA Quality Indicators in a Large Single-Center Surgical Breast Cancer Cohort from North Africa
Source: Cancers (Basel). 2026 Feb 25;18(5):731. doi: 10.3390/cancers18050731 (PMC12984865; doi:10.3390/cancers18050731)
Supplement: Supplementary file 1 [file cancers-18-00731-s001.zip › cancers-4095889-supplementary.pdf]

Long-Term Outcomes and EUSOMA Quality Indicators in a Large Single-Center Surgical Breast Cancer Cohort from North Africa

Table S1. Correlation of molecular subtypes with prognostic factors.

Variable	Luminal A	Luminal B	HER2 +	Triple -	p Value
**Age**	806	192	82	216	
< 40 Yrs	92 (11.5%)	25 (13.2%)	18 (22.2%)	36 (16.7%)	0.019
> 40 Yrs	714 (88.5%)	167 (86.8%)	64 (77.8%)	180 (83.3%)	
**Localisation**	795	190	82	211	
Right	374 (47.1%)	94 (49.4%)	43 (52.4%)	132 (62.5%)	
Left	389 (48.9%)	93 (49%)	35 (43.6%)	78 (37%)	0.001
Both	32 (4%)	3 (1.6%)	4 (4%)	1 (0.5%)	
**Number**	806	192	82	216	
Unifocal	678 (86.3%)	162 (84.4%)	71 (86.6%)	200 (92.6%)	0.058
Multifocal	110 (13.7%)	30 (15.6%)	11 (13.4%)	16 (7.4%)	
**Surgery**	806	192	82	215	
Conservatif	483 (60%)	101 (52.6%)	47 (57.3%)	142 (66.1%)	0.050
Radical	323 (40%)	91 (47.4%)	35 (42.7%)	73 (33.9%)	
**pT**	729	175	76	191	
pT1	252 (34.6%)	48 (27.4%)	16 (21.1%)	46 (24%)	
pT2	381 (52.2%)	103 (58.9%)	52 (68.4%)	126 (66%)	
pT3	90 (12.4%)	21 (12%)	7 (9.2%)	18 (9.6%)	0.020
pT4	6 (0.8%)	3 (1.7%)	1 (1.3%)	1 (0.5%)	
**pN**	765	185	77	202	
pN0	341 (44.6%)	60 (32.4%)	23 (29.9%)	111 (55%)	<0.001
pN+	424 (55.4%)	125 (67.6%)	54 (70.1%)	91 (45%)	
**SBR**	754	180	78	176	
SBR I	97 (12.9%)	11 (6.1%)	1 (1.3%)	3 (1.7%)	
SBR II	433 (57.4%)	75 (41.7%)	22 (28.2%)	46 (26.1%)	<0.001
SBR III	224 (29.7%)	94 (52.2%)	55 (70.5%)	127 (72.2%)	
**TNM Stage**	593	131	54	148	
I	138 (23.2%)	22 (16.8%)	3 (5.5%)	23 (15.6%)	
II	317 (53.5%)	67 (51.1%)	31 (57.5%)	99 (66.9%)	
III	125 (21.1%)	37 (28.2%)	17 (31.5%)	21 (14.2%)	0.002
IV	13 (2.2%)	5 (3.9%)	3 (5.5%)	5 (3.3%)	

For each EUSOMA quality indicator, the denominator was defined according to the original EUSOMA specifications (invasive disease only, DCIS only, or combined in invasive/DCIS populations, as applicable).

Table S2. A Two-Period Comparison of Quality Indicators in Breast Cancer Management.

Indicator	Percentage	Period I	Period II	Standard Minimum	Target
Proportion of women with breast cancer (invasive or in situ) who had a preoperative histologically or cytologically confirmed malignant diagnosis (B5 or C5).	29.6%	22.2%	37.1%	85%	95%
Proportion of invasive cancer cases for which the following prognostic/predictive parameters have been recorded: histological type, grading, PgR, ER, HER-2/neu, Proliferation index (Ki67)	97.7%	96.6%	98.9%	>96%	>98%
Time interval of 6 weeks, from the date of registration at oncology center to the date of surgery or start of other treatment	98.2%	97.6%	98.8%	80%	90%
Proportion of cancer cases examined preoperatively by MRI (excluding patients treated with PST)	1.65%	0.3%	3.4%	10%	NA
Proportion of patients treated with PST undergoing MRI (pre, during, post PST)	3.45%	0%	6.9%	60%	90%
Proportion of patients (invasive cancer only) who received a single (breast) operation for the primary tumor (excluding reconstruction)	98%	97.3%	98.7%	80%	90%
Proportion of patients (DCIS only) who received just one operation (excluding reconstruction)	79.5%	80%	79%	70%	90%
Proportion of patients with invasive breast cancer (M0) who received postoperative radiation therapy (RT) after surgical resection of the primary tumor and appropriate axillary staging/surgery in the framework of BCT	92%	90%	93.4%	90%	95%
Proportion of patients with involvement of axillary lymph nodes (pN2a) who received post-mastectomy radiation therapy to the chest wall and all (non-resected) regional lymph-nodes	**90%**	**92%**	**89%**	90%	95%
Proportion of patients with invasive cancer and clinically negative axilla who underwent sentinel lymph-node biopsy (SLNB) only	3.4%	2.7%	4.1%	90%	95%
Proportion of patients with invasive cancer who underwent sentinel lymph-node biopsy with no more than 5 nodes excised	**94.6%**	**100%**	**89.2%**	90%	95%
Proportion of patients with invasive breast cancer not greater than 3 cm who underwent BCT as primary treatment.	68.3%	66.4%	70.3%	70%	85%

Proportion of patients with non-invasive breast cancer not greater than 2 cm who underwent BCT	50%	50%	50%	80%	90%
Proportion of patients with endocrine sensitive invasive cancer who received endocrine therapy	**94.4%**	**93.1%**	**95.8%**	85%	90%
Proportion of patients with HER2 positive (IHC 3+ or in situ hybridisation positive FISH-positive) invasive carcinoma (T > 1 cm or N+) treated with chemotherapy who received adjuvant trastuzumab	89.4%	90.6-%	88.2%	85%	95%
Proportion of patients with HER2-positive invasive carcinoma treated with neoadjuvant chemotherapy who received neo-adjuvant trastuzumab	100%	100%	100%	90%	95%
Proportion of patients with inflammatory breast cancer (IBC) or locally advanced non-resectable ER-carcinoma who received neo-adjuvant chemotherapy	89.9%	85.7%	94.1%	90%	>95%

Table S3. Comparative table describing baseline clinicopathologic characteristics of patients included vs excluded in the survival analysis.

Parameter	Patients included 1695 (%)	Patients excluded 131 (%)	p
**Age**			
< 40 years	233 (13.7%)	21 (16%)	0.467
> 40 years	1462 (86.3%)	110 (84%)	
**Laterality**			
Right Breast	821 (48.4%)	60 (45.9%)	0.10
Left Breast	772 (45.6%)	56 (42.8%)	
Both Breasts	44 (2.5%)	8 (6.6%)	
Unknown	58 (3.5%)	6 (4.7%)	
**Number Of Tumor**			
Unifocal	1496 (88.3%)	114 (87%	0.673
Multifocal	199 (11.7%)	17 (13%)	
**Quadrant Distribution**			
QSE	362 (21.4%)	26 (19.7%)	0.282
Other Quadrant	884 (52.1%)	72 (55.1%)	
Unknown	449 (26.4%)	33 (25.2%)	
**Neoadjuvant Chemotherapy**			
Yes	118 (7%)	7 (5.3%)	0.47
No	1577 (93%)	124 (93.9%)	
**Surgery**			
Conservative	967 (57.1%)	77 (58.3%)	0.7
Radical	728 (42.9%)	54 (40.9%)	
**Lymph Node Dissection**			
Yes	1567 (92.4%)	119 (90.2%)	0.34
No	128 (7.6%)	13 (9.8%)	
**pT Classification**			
pT1	448 (26.5%)	29 (22.1%)	0.510
pT2	843 (49.8%)	65 (49.6%)	
pT3	170 (10%)	9 (6.8%)	
pT4	13 (0.7%)	0	
pTx	221 (13%)	28 (21.3%)	
**pN Classification**			
pN0	674 (39.7%)	50 (38.1%)	0.867
pN+	848 (50%)	65 (49.6%)	
pNx	173 (10.3%)	16 (12.2%)	
**Vascular Emboli**			
Yes	461 (27.2%)	34 (25.8%)	0.720
No	1234 (72.8%)	97 (74.2%)	

**Hormonal Receptor**			
Positif	1237 (72.9%)	90 (68.7%)	
Negatif	358 (21.3%)	33 (25.1%)	0.264
Unknown	100 (5.8%)	8 (6.2%)	
**HER-2**			
Positif	275 (16.2%)	17 (12.9%)	
Negatif	1024 (63.3%)	86 (65.6%)	0.262
Unknown	396 (23.3%)	28 (21.3%)	
**Biomolecular Classification**			
Luminal A	805 (47.4%)	58 (44.2%)	
Luminal B	192 (11.3%)	15 (11.4%)	
Triple Negatif	82 (4.8%)	2 (1.5%)	0.076
HER2+	216 (12.7%)	25 (19%)	
**TNM Stage**			
I	216 (12.7%)	11 (8.3%)	
II	616 (36.3%)	45 (34.3%)	
III	236 (7.18%)	19 (14.5%)	0.165
Iv	29 (1.7%)	0	
Unknown	598 (35.2%)	57 (43.5%)	
